# EXAMINING THE IMPACT OF RIDE-HAILING ON THE TRAVEL BEHAVIOR OF OLDER ADULTS ACROSS THREE CITIES

**DOI:** 10.1093/geroni/igae098.1311

**Published:** 2024-12-31

**Authors:** Minju Song

**Affiliations:** Portland State University, Portland, Oregon, United States

## Abstract

Due to age-related physical and cognitive changes, older drivers may eventually find themselves unable to continue driving, when finding alternative transportation modes becomes a major concern. Ride-hailing services like Uber and Lyft have emerged as potential solutions to this issue. Nevertheless, given the dearth of available data and the recently introduced transportation systems, limited research exists on ride-hailing usage of older adults. This paper aims to examine the factors influencing ride-hailing adoption among older adults by comparing different age groups across three metropolitan areas: Chicago, Minneapolis, and Seattle area. Using regional survey data collected in 2019, we estimate logistic regression models by age groups to examine notable characteristics of older adults utilizing ride-hailing. In follow-up, we use a multilevel logistic regression model to explore the hierarchical relationships between regional, household, and individual levels. Findings show that ride-hailing services are more used by younger adults (18-64) compared to individuals over 65 years, indicating a potential technological familiarity gap between age groups. The built environment has a significant association with ride-hailing adoption, particularly in urban areas with transit infrastructure and higher population density. While ride-hailing has the potential to enhance the social inclusion and independence of older people who stop driving, ride-hailing is not accessible for older people as much as other age groups. Thus, this study underscores the importance of continued investment and research in ride-hailing services, advocating for policymakers and service providers to consider older adults’ needs in transportation planning.

